# Supplementary material for: Mechanisms governing the pioneering and redistribution capabilities of the non-classical pioneer PU.1
Source: Nat Commun. 2020 Jan 21;11:402. doi: 10.1038/s41467-019-13960-2 (PMC6972792; doi:10.1038/s41467-019-13960-2)
Supplement: Supplementary file 7 — Source data [file 41467_2019_13960_MOESM7_ESM.zip › Source_Data/FigureS2/FigureS2B_MotifScanOutput/KmeansCluster8/homerResults/motif4.similar.html]

motif4

## Information for motif4

C
T
A
G
A
G
C
T
A
C
G
T
A
G
C
T
A
G
T
C
A
G
T
C
A
C
G
T
  
Reverse Opposite:  

C
G
T
A
A
C
T
G
A
C
T
G
C
T
G
A
C
G
T
A
T
C
G
A
A
G
T
C
  

|  |  |
| --- | --- |
| p-value: | 1e-88 |
| log p-value: | -2.035e+02 |
| Information Content per bp: | 1.869 |
| Number of Target Sequences with motif | 674.0 |
| Percentage of Target Sequences with motif | 44.58% |
| Number of Background Sequences with motif | 9582.0 |
| Percentage of Background Sequences with motif | 21.11% |
| Average Position of motif in Targets | 100.4 +/- 55.9bp |
| Average Position of motif in Background | 101.2 +/- 74.5bp |
| Strand Bias (log2 ratio + to - strand density) | -0.1 |
| Multiplicity (# of sites on avg that occur together) | 1.20 |
| Motif File: | file (matrix) reverse opposite |

### Similar de novo motifs found

|  |  |  |  |  |  |  |  |
| --- | --- | --- | --- | --- | --- | --- | --- |
| Rank | Match Score | Redundant Motif | P-value | log P-value | % of Targets | % of Background | Motif file |
| 1 | 0.824 | G C T A G T A C C T A G A G C T A G C T A C G T A G T C A G T C A C G T C T A G | 1e-16 | -37.071747 | 3.24% | 0.71% | motif file (matrix) |
| 2 | 0.807 | C T A G A C G T C G A T A C G T A G T C A G T C A C G T A C T G A C G T C G A T | 1e-14 | -34.068895 | 2.18% | 0.34% | motif file (matrix) |
